# Anatomic Variations of the Right Hepatic Duct: Results and Surgical Implications from a Cadaveric Study

**DOI:** 10.1155/2012/838179

**Published:** 2012-09-29

**Authors:** Theodoros Mariolis-Sapsakos, Vasileios Kalles, Konstantinos Papatheodorou, Nikolaos Goutas, Ioannis Papapanagiotou, Ioannis Flessas, Ioannis Kaklamanos, Demetrios L. Arvanitis, Evangelos Konstantinou, Markos N. Sgantzos

**Affiliations:** ^1^Laboratory of Anatomy, School of Nursing, University of Athens, 123 Papadiamantopoulou Street, Goudi, 11527 Athens, Greece; ^2^Laboratory of Forensics and Toxicology, Medical School, University of Athens, 75 Mikras Asias Street, Goudi, 11527 Athens, Greece; ^3^Department of Anatomy, Medical School, University of Thessaly, New Medical School Building, Viopolis, 1400 Larissa, Greece

## Abstract

*Purpose*. Thorough understanding of biliary anatomy is required when performing surgical interventions in the hepatobiliary system. This study describes the anatomical variations of right bile ducts in terms of branching and drainage patterns, and determines their frequency. *Methods*. We studied 73 samples of cadaveric material, focusing on the relationship of the right anterior and posterior segmental branches, the way they form the right hepatic duct, and the main variations of their drainage pattern. *Results*. The anatomy of the right hepatic duct was typical in 65.75% of samples. Ectopic drainage of the right anterior duct into the common hepatic duct was found in 15.07% and triple confluence in 9.59%. Ectopic drainage of the right posterior duct into the common hepatic duct was discovered in 2.74% and ectopic drainage of the right posterior duct into the left hepatic duct in 4.11%. Ectopic drainage of the right anterior duct into the left hepatic ductal system and ectopic drainage of the right posterior duct into the cystic duct was found in 1.37%. *Conclusion*. The branching pattern of the right hepatic duct was atypical in 34.25% of cases. Thus, knowledge of the anatomical variations of the extrahepatic bile ducts is important in many surgical cases.

## 1. Purpose

Anatomic variations of the extrahepatic bile ducts are important during surgical procedures such as laparoscopic cholecystectomy, liver resection (hepatectomy, segmentectomy), and living donor transplantation [1, 2]. It has been shown that the frequency of bile duct injuries occurring during laparoscopic cholecystectomies is twice as high as those occurring during open cholecystectomies [3]. Furthermore, evaluation of the biliary anatomy is essential before hepatic lobectomy or segmentectomy, as inaccurate determination of existing biliary anatomic variations may potentiate ligature or section of aberrant ducts, leading to major complications such as leakage or atrophy of the residual liver [2]. Therefore, it is apparent that thorough knowledge and successful detection and recognition of such anatomic variations can lead to decreased morbidity and mortality rates during hepatobiliary surgery.

Although several methods, like CT or MR cholangiograms, have become the modality of choice for noninvasive evaluation of abnormalities of the biliary tract, they are not routinely used in preoperative imaging evaluation of patients undergoing common procedures such as laparoscopic cholecystectomy. Also, several uncommon—and usually more complicated—anatomic variations of the bile duct have been described. Thus, an accurate knowledge of the anatomy of the extrahepatic biliary tree and its variants' frequency is critical in order to be highly suspicious while operating in that region.

The purpose of this study is to describe anatomic variations of the right hepatic duct in terms of the branching pattern and to determine the frequency of each variation. To this end, we studied cadaveric hepatobiliary material.

## 2. Methods

The material we used in our study consists of 73 samples from cadaveric incisions that took place at the Laboratory of Forensics and Toxicology of the University of Athens during the period of October 2010 to December 2011. The study received appropriate approval from the University of Athens' ethics committee. Our material derives from 35 males and 38 females. Samples with cirrhosis, hepatobiliary cancer, hepatobiliary injuries, and previous operations in the liver or the biliary system were excluded from the study.

The study was focused on the anatomical relation of the right anterior and posterior segmental branches to the formation of the right hepatic duct and on the variants of their drainage, as the knowledge of the anatomical variations of the right hepatic duct and its main branches is critical for successful surgical interventions that involve the hepatobiliary system.

## 3. Results

**Figure 1:**
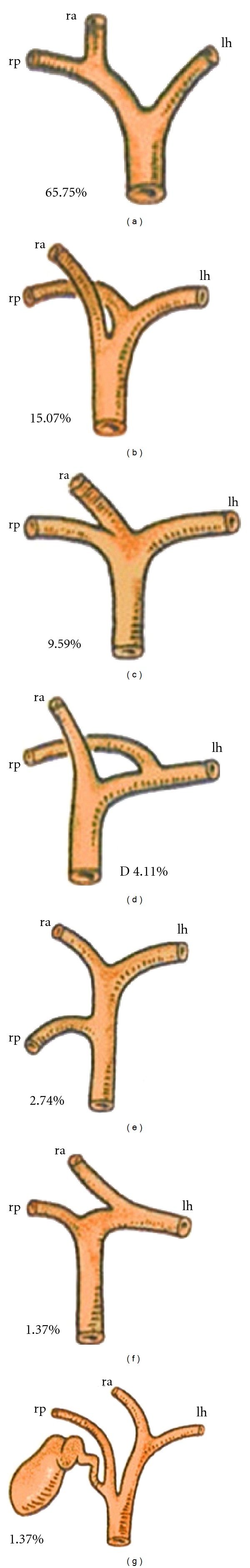
Main variations of the right hepatic duct in Greek cadavers. (a) Typical anatomy of the right hepatic duct (*n* = 48, 65.75%). (b) Ectopic drainage of the right anterior duct into the common hepatic duct (*n* = 11, 15.07%). (c) Triple confluence, the common hepatic duct receives independently the right anterior duct, the right posterior duct, and the left hepatic duct (*n* = 7, 9.59%). (d) Ectopic drainage of the right posterior duct into the left hepatic ductal system (*n* = 3, 4.11%). (e) Ectopic drainage of the right posterior duct into the common hepatic duct (*n* = 2, 2.74%). (f) Ectopic drainage of the right anterior duct into the left hepatic ductal system (*n* = 1, 1.37%). (g) Ectopic drainage of the right posterior duct into the cystic duct (*n* = 1, 1.37%). ra: right anterior; rp: right posterior; lh: left hepatic.

The right hepatic duct drains the segments of the right liver lobe (V–VIII) and has two major branches: the right posterior branch draining the posterior segments, VI and VII, and the right anterior duct draining the anterior segments, V and VIII. The right posterior duct has an almost horizontal course, whereas the right anterior duct tends to have a more vertical course. The right posterior duct usually runs posterior to the right anterior duct and fuses it from a left (medial) approach to form the right hepatic duct. The left hepatic duct is formed by segmental tributaries draining segments II–IV. The common hepatic duct is formed by fusion of the right hepatic duct, which is usually short, and the left hepatic duct. The cystic duct classically joins the common hepatic duct below the confluence of the right and left hepatic ducts [4]. This typical anatomy of the right hepatic duct and of the common hepatic duct formation was encountered in 48 of our samples (65,75%) (Figure 1(a)).

According to Couinaud's nomenclature, the most common anatomic variants in the branching of the biliary tree described involve the right posterior duct and its fusion with the right anterior or the left hepatic duct. In our study, ectopic drainage of the right anterior segmental duct into the common hepatic duct was recognized in 11 out of the 73 samples (15.07%) (Figure 1(b)).

Another variant of the main hepatic biliary branching, the so-called triple confluence, is an anomaly characterized by simultaneous emptying of the right posterior duct, right anterior duct, and left hepatic duct into the common hepatic duct. In patients with this variant, the right hepatic duct is virtually nonexistent. The specific variation was encountered in seven of our samples (9.59%) (Figure 1(c)).

Drainage of the right posterior hepatic duct into the left hepatic duct before its confluence with the right anterior duct is another relatively common anatomic variant of the biliary system and it presented in three of the samples (4.11%) (Figure 1(d)). The direct drainage of the right posterior duct into the common hepatic duct is a variant also known as the aberrant hepatic duct and was present in two of our samples (2.74%) (Figure 1(e)). Finally, we found one of each (1,37%) of some even less common variants of the biliary tree, like ectopic drainage of the right anterior duct into the left hepatic ductal system, and ectopic drainage of the right posterior duct into the cystic duct (Figures 1(f) and 1(g)).

## 4. Discussion

 Thorough knowledge of anatomical variations is of key importance during surgical procedures, especially when it comes to anatomic areas with high rates of variations, such as the hepatobiliary system. Many anatomical studies have been conducted in order to determine the specific anatomical variations, using cadaveric material, intraoperative data, or imaging such as ultrasonography and magnetic resonance cholangiography [2, 5, 6].

Awareness of possible variations of the biliary system is crucial in liver transplantations [7, 8]. Preoperative or intraoperative identification of the atypical or anomalous ducts and appropriate tailoring of the surgical technique are important in order to avoid serious postoperative complications [9–11].

A good example of uncommon but yet important variations of the bile duct are the aberrant and the accessory bile ducts. In the clinical context, familiarity with these two different entities is important because an aberrant bile duct is the only bile duct draining a particular hepatic segment, whereas an accessory one is an additional bile duct draining the same area of the liver. Therefore, when performing a left hepatectomy in a living related transplant donor, it is of great importance to recognize aberrant drainage of the right posterior duct or right anterior duct into the left hepatic duct, as the ligation of these ducts will produce biliary cirrhosis of segments VI and VII, or segments V and VIII, respectively [12].

Anatomic variations of the biliary tract are often accompanied by variations in the portal venous system and the hepatic arterial system, which are also important in liver resections and transplants, and several authors have examined the relationship between them [13–17]. More specifically, portal venous anomalies have been demonstrated to significantly correlate with anomalous biliary drainage [14, 16, 17], especially in the right lobe [17], and this concordance is likely to be related to the embryology of the biliary tree itself [18]. However, in another study, Macdonald et al., who investigated the relationship between vascular and biliary anatomy in a relatively small sample of living liver donors, concluded that this association was not significant [15], while Lee et al. found no significant association between hepatic arterial variants and biliary variants [16]. Finally, most investigators seem to support the hypothesis that certain vascular and biliary variants are not associated with each other [14–16]. 

Anatomic factors, including the presence of anomalies of the cystic duct and the hepatic ducts, constitute one of the major causes of bile duct injuries after laparoscopic cholecystectomy [3, 19]. Additionally, the need for altering the surgical technique due to an anatomic variation is not uncommon here either [20]. In patients with drainage of the cystic duct into the left side of the common hepatic duct, it is generally preferable to leave a long cystic duct remnant instead of dissecting the cystic duct up to the left side of the common hepatic duct [19]. 

Our findings in the Greek cadavers are, in general, consistent to those reported in similar studies, although with a few dissimilarities (Table 1). The normal biliary anatomy has been reported to be present in 52,9%–58% of the population [12, 21, 22], slightly less frequently than in our sample series. Our rates of ectopic drainage of the right anterior segmental duct in the common hepatic duct are almost identical to the already reported rates [22]. Although anomalous drainage of the right posterior segmental duct into the left hepatic duct, which is described as the most common atypical anatomy, with rates from 13% to 19% [12, 21–23], seemed to be less frequent in our cadavers' sample. On the other hand, the triple confluence presence rate in our study is comparable to the 11% to 12% reported in other studies [12, 21, 22]. Direct drainage of the right posterior duct into the common hepatic duct, reportedly present in 4% to 5% of the population [12, 22], was also encountered in similar rates in our study. Finally, rates of rare variants of biliary branching such as ectopic drainage of the right anterior duct into the left hepatic duct or ectopic drainage of the right posterior duct into the cystic duct were similar to those already reported by other investigators [13, 21, 22]. 

## 5. Conclusion

In summary, atypical branching patterns of the right hepatic duct were found in 34,25% of cases. The two most common variations were the ectopic drainage of the right anterior duct into the common hepatic duct (15,07%), and trifurcation of the right anterior segmental duct, right posterior segmental duct, and left hepatic duct (9,59%). 

Both intra- and extrahepatic biliary anatomies are complex with the existence of many common and uncommon anatomic variations. The increasing complexity of hepatic surgical procedures and biliary interventions, necessitate widespread and appropriate knowledge of these anatomic variations, in order to avoid possible complications and help achieve the most effective result.

## Figures and Tables

**Table 1:** Frequency of anatomic variations of the right hepatic ductal system in Greek cadavers and reported presence in existing studies [12, 13, 21–23].

Anatomical variation	Samples (*n*)		Samples (%)		Reported presence (%)
	Males	Females	Males	Females
Typical anatomy of the right hepatic duct	24	24	68,57	63,15	52,9–58
Total	48		65,75	

Ectopic drainage of the anterior duct into the common hepatic duct	5	6	14,28	15,78	16
Total	11		15,07	

Triple confluence	3	4	8,57	10,52	11-12
Total	7		9,59	

Ectopic drainage of the right posterior duct into the left hepatic ductal system	1	2	2,85	5,26	13–19
Total	3		4,11	

Ectopic drainage of the right posterior duct into the common hepatic duct	1	1	2,85	2,63	4,5
Total	2		2,74	

Ectopic drainage of the right anterior duct into the left hepatic ductal system	1	0	2,85	0	1
Total	1		1,37	

Ectopic drainage of the right posterior duct into the cystic duct	0	1	0	2,63	2
Total	1		1,37	

Total	35	38	100	100	
	73			
